# Gap filling between GRACE and GRACE-FO missions: assessment of interpolation techniques

**DOI:** 10.1007/s00190-024-01917-3

**Published:** 2024-11-23

**Authors:** Hugo Lecomte, Severine Rosat, Mioara Mandea

**Affiliations:** 1 ITES, Université de Strasbourg (CNRS UMR7063), 5 Rue Descartes, 67084 Strasbourg, France; 2 LEGOS, Université de Toulouse, 31400 Toulouse, France; 3 Centre Nationale d’Etudes Spatiales, CNES, 75001 Paris, France

**Keywords:** Variable gravity field, Gap filling, Interpolation, GRACE, Swarm

## Abstract

We propose a benchmark for comparing gap-filling techniques used on global time-variable gravity field time-series. The Gravity Recovery and Climate Experiment (GRACE) and the GRACE Follow-On missions provide products to study the Earth’s time-variable gravity field. However, the presence of missing months in the measurements poses challenges for understanding specific Earth processes through the gravity field. We reproduce, adapt, and compare satellite-monitoring and interpolation techniques for filling these missing months in GRACE and GRACE Follow-On products on a global scale. Satellite-monitoring techniques utilize solutions from Swarm and satellite laser ranging, while interpolation techniques rely on GRACE and/or Swarm solutions. We assess a wide range of interpolation techniques, including least-squares fitting, principal component analysis, singular spectrum analysis, multichannel singular spectrum analysis, auto-regressive models, and the incorporation of prior data in these techniques. To inter-compare these techniques, we employ a remove-and-restore approach, removing existing GRACE products and predicting missing months using interpolation techniques. We provide detailed comparisons of the techniques and discuss their strengths and limitations. The auto-regressive interpolation technique delivers the best score according to our evaluation metric. The interpolation based on a least-squares fitting of constant, trend, annual, and semi-annual cycles offers a simple and effective prediction with a good score. Through this assessment, we establish a starting benchmark for gap-filling techniques in Earth’s time-variable gravity field analysis.

## Introduction

Our planet is a dynamic system where various processes affect the distribution of mass in the Earth and produce variations in its gravity field on a wide range of spatial and temporal scales. Measurements of the Earth’s gravity field from space make it possible to understand the processes that shift masses within the Earth, and on and above its surface. For more than 20 years two emblematic missions have measured nearly continuously the gravity field.

The Gravity Recovery and Climate Experiment (GRACE) satellite mission and its follow-on, GRACE-Follow On (GRACE-FO), enable precise measurements of Earth’s time-variable gravity field (Tapley et al. 2004; Landerer et al. 2020). For the sake of concision, the notation GRACE(-FO) refers to both GRACE and GRACE-FO missions. GRACE(-FO) measurements have been used to study mass variations in various Earth’s system components, including regional to global scale terrestrial water storage (TWS) changes, mass changes of polar ice sheets and glaciers, global mean ocean mass changes, solid Earth mass changes, deep Earth’s signals, and others (Chen et al. 2022a).

Time-variable gravity field solutions of GRACE(-FO) are delivered as multiple products with a monthly temporal resolution. In the following, the term “product” refers to a Stokes coefficients estimation of the gravity field for one month (corresponding to Level 2), while the term “solution” refers to an ensemble of products delivered by one processing center. The first product of the GRACE mission covers April 2002 and the last product covers June 2017. The GRACE-FO mission was launched in May 2018; its first product covers June 2018 up to the present. An 11-month gap without any product exists then between the two missions. Throughout the lifetimes of the two missions, testing procedures and technical problems have led to a total of 22 missing months (excluding the 11-month gap) as of the time of this article.

In order to continue the measurement of Earth’s time-variable gravity field, a new satellite mission is needed prior to the end of the GRACE-FO mission. The next satellite gravimetry mission is called GRACE-Continuity and is also referred to as the Mass Change Mission (MCM). The anticipated launch of GRACE-Continuity is set for 2028 (Landerer 2023), hopefully before the end of the GRACE-FO mission. However, the possibility of a gap between GRACE-FO and the following satellite mission cannot be omitted even if it would not be optimal.

The missing months in GRACE(-FO) solutions represent a challenge for diverse applications and users. The uneven sampling of the gravity-field time-series can introduce biases in trend and inter-annual cycle estimations (Santamaría-Gómez and Ray 2021; Yi and Sneeuw 2021). The spectral analysis of irregularly sampled data is a problem in widespread applications (Babu and Stoica 2010). Monitoring sub-monthly and monthly events, such as floods or earthquakes, becomes impossible if gaps occur during the time period of the event. For instance, accurately quantifying terrestrial water storage changes requires a procedure for interpolating GRACE estimates when GRACE or GRACE-FO data are unavailable (Argus et al. 2022). Some users might choose to accept the biases created by these gaps to minimize modifications to the time-series, while others might apply simple or sophisticated methods to fill in the data gaps and reduce these biases. In the near future, product producers might also consider distributing Level 3 or Level 4 data without gaps that are easier to handle for end-users without strong knowledge of the GRACE(-FO) solutions. For example, products with a flag indicating the possible interpolation process.

The time-variable gravity field can be recovered by various spatial techniques other than GRACE(-FO). Precise orbit determination of low Earth’s orbit satellites can be used to generate monthly gravity field solutions (Chen et al. 2022b). Since the 1980 s, satellite laser ranging (SLR) delivers products on the lowest-degree coefficients of the Earth’s gravity field (Couhert et al. 2020). Weigelt (2019) proposes to use gravity field products based on high-low satellite-to-satellite tracking and SLR to bridge the gap between the two GRACE(-FO) missions. Löcher and Kusche (2020) compute empirical orthogonal functions of the monthly gravity field based on GRACE(-FO) observations and extend the estimation of these functions with SLR data for low degrees and on months with missing GRACE(-FO) products. These two determinations of the gravity field can be used to fill the gaps, although they are noisier than GRACE(-FO) products. They can also be used as an external reference to be compared with gap-filling techniques assessed in this work.

**Fig. 1:**
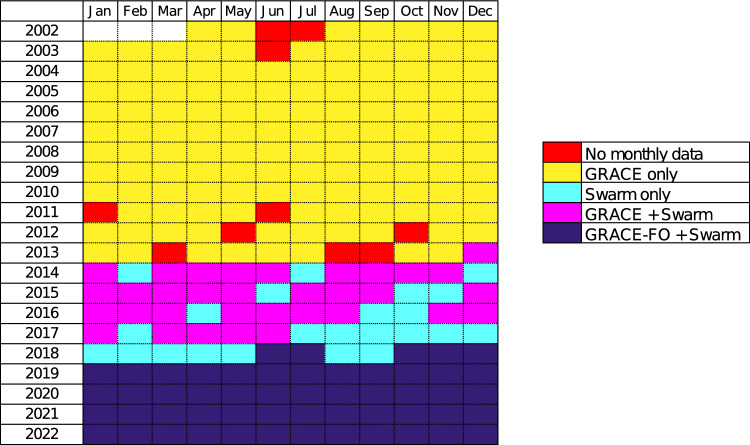
Monthly calendar of the availability of GRACE(-FO) and Swarm time-variable gravity field products

Another mission providing the needed measurements to describe the gravity field is Swarm. This mission is ESA’s first constellation mission for Earth Observation program and consists of three identical satellites, launched on November 22, 2013, into a near-polar orbit. Swarm provides high-precision and high-resolution measurements of the Earth’s magnetic field, complemented by precise navigation, accelerometer, and electric field measurements. Important for our study is the possibility offered by Swarm to deliver stand-alone time-variable gravity field solutions operable at low-degrees ($\le$ 12) (Friis-Christensen et al. 2006). The orbital perturbations of the three Swarm satellites enable us to measure the gravity field (Jäggi et al. 2016; Lück et al. 2018), although the resulting products contain more noise than those of GRACE(-FO). The first gravity field variation product of the Swarm mission covers December 2013, and thereafter, the products are available over the 11-month gap between GRACE and GRACE-FO (Fig. 1).

Multiple works available in the literature propose different techniques to fill the missing months in GRACE(-FO) solutions. The aim of this paper is to reproduce, adapt, and compare a specific category of these techniques. We restrict our comparison to techniques that estimate global monthly products on an Earth-wide scale. Some other works, not reproduced here, propose predictions at the local scale of hydrological basins. A large number of them are referenced in the review proposed by Bimal et al. (2022). The Earth-wide techniques can be categorized in three groups (as proposed in Gu et al. (2024)): data-driven methods, satellite-monitoring methods, and interpolation methods. In our comparison, we narrow our focus to satellite-monitoring and interpolation methods. Data-driven methods encompass machine-learning techniques that use non-gravitational data related to surface displacement (e.g., Rietbroek et al. 2014 with Global Positioning System (GPS)/ Global Navigation Satellite System) and/or to climate variables (e.g., Humphrey et al. 2017; Li et al. 2020 with precipitation, temperature). However, comparing data-driven methods requires manipulating an excessively large number of datasets. This excludes neural networks predictions from our comparison, as they use GRACE(-FO) solution along with various other variables, such as climate data, to have a sufficient training dataset (e.g., Sun et al. 2020; Mo et al. 2022).

Satellite-monitoring methods are based on time-variable gravity field solutions from SLR, Swarm or high-low satellite-to-satellite tracking. These solutions can serve as substitutes for missing GRACE(-FO) months, but they can also be used in conjunction with interpolation methods to estimate interpolated products. The estimation of the interpolated months can be made through the fitting of constant, trend, annual, and semiannual (CTAS) components using least-squares methods. Alternatively, sophisticated approaches involve decomposing the time-series using principal component analysis (PCA) (Gu et al. 2024), or auto-regressive (AR) model (Lenczuk et al. 2022) techniques. The interpolation can be based on GRACE(-FO) solution and on other satellite solutions (Lück et al. 2018; Forootan et al. 2020; Richter et al. 2021). Other interpolation methods are exclusively based on GRACE(-FO) solution. These include decomposing the time-series using singular spectrum analysis (SSA) (Yi and Sneeuw 2021) or multichannel SSA (MSSA) (Wang et al. 2021) techniques. Within the scope of our assessment, some methods also incorporate a prior model to reduce the data variance. Richter et al. (2021) achieve this by removing CTAS components from the data. Alternatively, the use of a hydrological loading model is proposed by Lenczuk et al. (2022) and Gu et al. (2024) for the same purpose.

With this panel of gap-filling techniques, achieving consensus on the most optimal approach remains elusive. This article has a pioneering role by establishing a benchmark with the first inter-comparison of numerous gap-filling techniques using satellite-monitoring and interpolation methods on a global scale. In this context, a benchmark refers to a standardized evaluation to compare and assess the performance of gap-filling techniques. This article distinguishes itself from prior works, such as the review by Bimal et al. (2022), which explores numerous techniques but focuses on hydrological basins and does not propose a unified metric to compare the results of different studies. Similarly, the work of Qian et al. (2022) provides a more in-depth comparison but only for three techniques, also at large spatial scales. To assess and compare the different techniques, we first introduce the datasets employed in our study. We outline our methodology based on a remove-and-restore approach. This ensures the comparison of technique estimations with real data by removing existing GRACE products from the dataset and predicting these removed months. Then, we present in detail the techniques that are compared here, pointing out the differences between our implementation and the original methodologies. Finally, we conduct a comparison, discussing the techniques alongside their associated statistical results in our assessment.

## Data and methodology

### Spherical harmonics (SH)

For the self-consistency of the paper we introduce the classical way to describe the Earth’s gravity field. The gravitational potential $V(\lambda , \phi )$ at longitude $\lambda$ and co-latitude $\phi$ at the Earth’s surface (mean radius $R=6.371 \times 10^6~$m) can be expressed in a spherical harmonics (SH) expansion as

1 $$\begin{aligned} V(\lambda , \phi )= &  - \frac{GM}{R}\nonumber \\  &  \left[ \sum _{l=0}^{\infty } \sum _{m=0}^{l} \left( C_{l,m} \cos m\lambda \, + \, S_{l,m} \sin m\lambda \right) \bar{P}_{l,m} (\cos \phi ) \right] ,\nonumber \\ \end{aligned}$$

where $M=5.972\times 10^{24}~$kg is the Earth’s mass, *G* $= 6.67430 \times 10^{-11}\,\text {m}^3\,\text {kg}^{-1}\,\text {s}^{-2}$ is the gravitational constant, $C_{l,m}$ and $S_{l,m}$ are the Stokes coefficients (dimensionless) of degree *l* and order *m*, and $\bar{P}_{l,m} (\cos \theta )$ are normalized associated Legendre polynomials (Wahr et al. 1998).

Assuming that mass variations in the Earth system are caused by surface processes, the time-variable gravity field can be represented as changes in surface mass expressed in equivalent water height (EWH), $\Delta H_w(\lambda , \phi )$, as

2 $$\begin{aligned} \Delta H_w(\lambda , \phi )= &  \frac{R \bar{\rho }}{3 \rho _w} \sum _{l=0}^{\infty } \sum _{m=0}^{l} \frac{2l + 1}{1 + k_l}\nonumber \\  &  \left[ \Delta C_{l,m} \cos (m \lambda ) + \Delta S_{l,m} \sin (m \lambda ) \right] \bar{P}_{l,m}(\cos \phi ), \nonumber \\ \end{aligned}$$

where $\bar{\rho }= 5515~\text {kg}\,\text {m}^{-3}$ is the mean density of the Earth, $\rho _w = 1000~\text {kg}\,\text {m}^{-3}$ is the density of water, and $k_l$ is the load Love number of degree *l* (Wahr et al. 1998).

### Time-variable gravity field products

#### GRACE(-FO)

Various analysis centers deliver monthly time-variable gravity field products in SH representation, each using an independent processing strategy. We use the combined GRACE(-FO) monthly gravity fields solution provided by the International Combination Service for Time-variable Gravity Fields (COST-G) center (Meyer et al. 2020) to cover the period from April 2002 to December 2022. This solution is estimated from a weighted combination of the SH solutions from various centers using variance component estimation (VCE) (Jean et al. 2018). It results from the combination of the AIUB-RL02, GFZ-RL06, GRGS-RL04, ITSG-GRACE2018, and CSR-RL06 solutions for the GRACE period and AIUB-GRACEFO_op, GFZ-RL06, GRGS-RL05, ITSG-Grace_op, LUH-GRACE-FO, CSR-RL06, and JPL-RL06 for the GRACE-FO period. This combination of solutions has the advantage to reduce the noise level of the time-series compared to the other stand-alone solution as well as the individual biases (Jäggi et al. 2020).

We use COST-G with the SH time-series corrected such that the average of each coefficient is null. This average is computed over the available months of the COST-G solution between April 2002 and December 2022. As recommended, Technical notes TN-14 solution based on SLR data is used to correct $C_{2,0}$ (Loomis et al. 2019) and $C_{3,0}$ after October 2016 (Loomis et al. 2020). Geocenter coefficients $C_{1,0}$, $C_{1,1}$, and $S_{1,1}$ are not included as they cannot be measured accurately by GRACE(-FO). COST-G products are corrected from the atmospheric and oceanic loading by the atmosphere and ocean de-aliasing Level 1B (AOD1B) model (Dobslaw et al. 2017). To be consistent with the Swarm solution we use SH coefficients from degree 2 to degree 12. However, we also consider the SH up to degree 40 after applying a Gaussian filter with 400 km radius, as detailed in the appendix.

As we truncate the SH representation at degree 12, we choose a SH and not a mascon solution. SH solutions are global, whereas mascons are designed to access higher spatial resolution with pre-established grids that are an a priori of the mass distribution (Scanlon et al. 2016). For the sake of comparing gap-filling techniques, the choice of the GRACE(-FO) time-series is not important. Changing from COST-G to another GRACE(-FO) solution does not significantly change the final results (not shown here).

#### Swarm

The Swarm time-variable gravity field is also a weighted combined solution using VCE generated by COST-G (Encarnacao et al. 2019). It results from the combination of individual solutions from the AIUB, ASU, IfG, and OSU centers, and it spans the period from December 2013 to December 2022. This combination also reduces the noise level of the time-series in comparison to the other stand-alone solution (Teixeira da Encarnação et al. 2020).

Swarm products have large root-mean-square (RMS) differences with GRACE products in 2014 attributed to a maximum in solar activity and a problem with the Global Positioning System (GPS) receivers’ onboard software (van den IJssel et al. 2016; Dahle et al. 2017). To reduce the effect of this low quality, we consider the Swarm products between April 2015 and December 2022. The SH decomposition of Swarm products goes up to degree 40. However, we only keep SH coefficients from degree 2 to degree 12, as they contain most of the geophysical signal recovered by Swarm; moreover, higher degrees are affected by noise (Teixeira da Encarnação et al. 2020).

Swarm products are corrected from sub-monthly atmospheric and oceanic loading effects using the AOD1B as for GRACE(-FO) products. In order to reduce the biases between GRACE(-FO) and Swarm, each SH coefficient of Swarm is corrected from the monthly mean of the difference between the two solutions. Because of this correction, our assessment does not evaluate the capacity of the gap-filling techniques to correct these potential biases.

### Hydrological loading model

Hydrological loading models can be used to reduce the variance of the time-variable gravity field caused by hydrological surface loading. In the following, we use the Global Land Data Assimilation System (GLDAS) Catchment Land Surface Model 2.1 (Rodell et al. 2004) that solves the vertical water mass balance but does not account for the lateral fluxes. We produce an average of GLDAS variations for each month in SH coefficients up to degree 12. The model might then be converted to a grid with a spatial resolution of $1^o$ per $1^o$. The gravitational potential changes induced by hydrological mass redistribution and loading (Newtonian attraction and mass redistribution associated with elastic deformation) are computed as detailed in Petrov and Boy (2004) and Gegout et al. (2010). The permanently ice-covered areas have been masked out as the model does not include ice sheets (Rodell et al. 2004).

### Methodology to compare gap-filling techniques

**Fig. 2:**
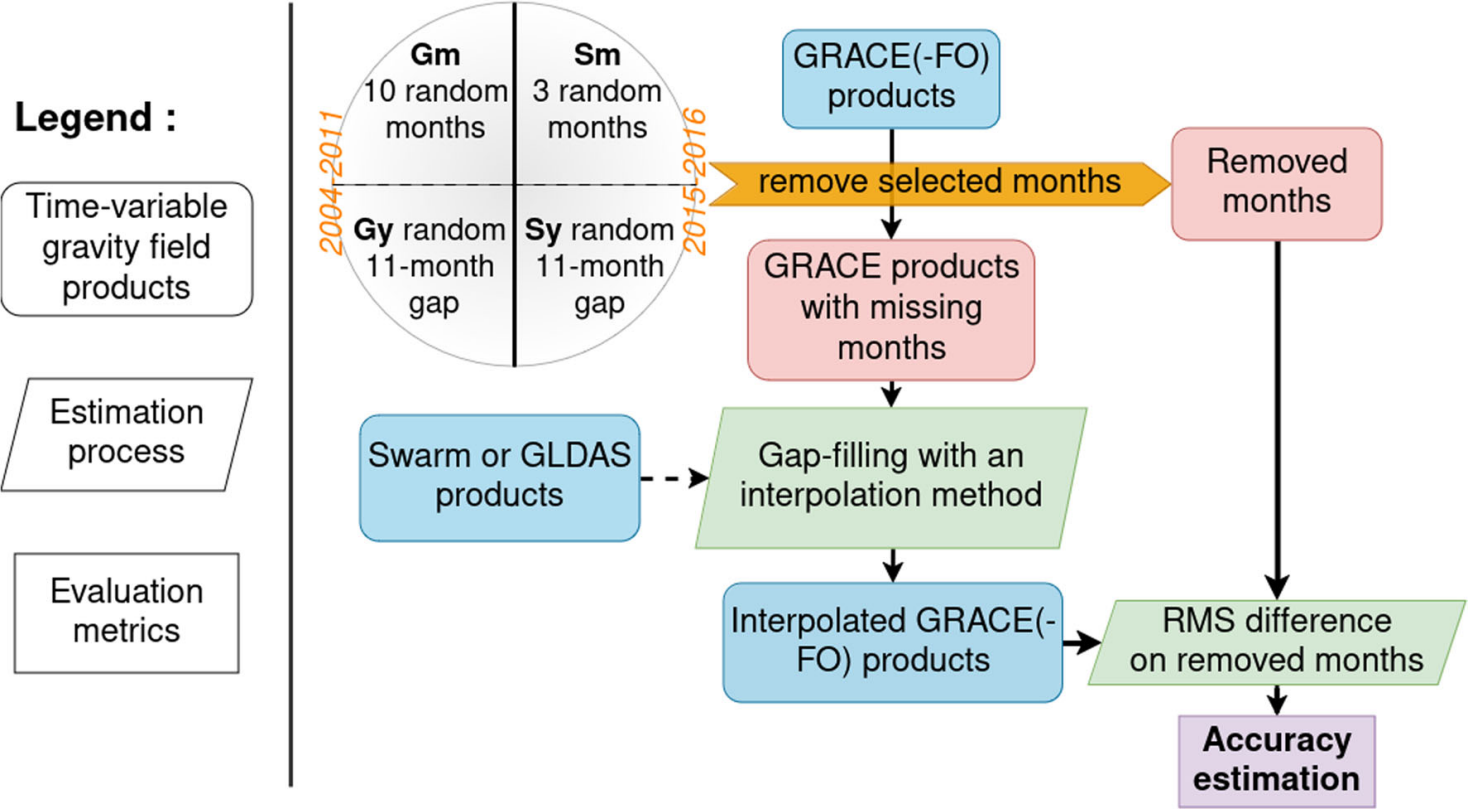
Schematic workflow of the methodology to assess gap-filling techniques efficiency

In order to compare and evaluate various gap-filling techniques for interpolating missing months in a solution, a systematic methodology is applied. This methodology proposes a benchmark to assess the different approaches capabilities to predict the content of missing month(s). To do this, we apply a remove-and-restore approach in which we I) randomly remove the contents of certain GRACE months, II) run a gap-filling technique to predict the data for these months, and III) compare them with the removed original time-segment. This process is repeated a certain number of times to retrieve some statistics (bootstrapping). The whole workflow is schematized in Fig. 2.

With this methodology, we employ GRACE as a reference to evaluate the gap-filling techniques. This choice can be questioned as other gravity field products exist. However, SLR solutions cannot, currently, be exploitable at degrees higher than 5 and the accuracy of a Swarm solution does not stand the comparison with a GRACE(-FO) solution. Cross-validation methods based on the sea level change or other independent data can also be considered, and we discuss these aspects afterward.

#### Evaluation metrics

The difference between the removed and the restored months is quantified as the RMS difference, which is a measure of the difference between two values.

3 $$\begin{aligned} \mathrm {RMS~difference} = \sqrt{ \frac{\sum _{i} [x_i - y_i]^2 }{n} }, \end{aligned}$$

where $x_i$ is the observed value at the *i*-th data (through time and/or latitude, longitude), and $y_i$ is the predicted value at the *i*-th data. The variable *n* denotes the total number of data.

The estimation of this metric can be made on various forms of data. When the products are expressed as SH, the RMS difference of each coefficient over time highlights which coefficients are the best retrieved. Once we project the products on a grid in EWH ($1^\circ \times 1^\circ$ degrees), we can compute the total RMS difference between the grids, weighted by the cosine of the latitude. We can also create a map of the RMS differences by calculating the RMS difference at each point of the grid.

The total RMS difference between the grids, weighted by latitudes, provides an estimation of the accuracy of each technique in EWH. Alongside this, the RMS difference for each SH coefficient or each spatial point allows us to gather more details on which coefficients or spatial regions are the most problematic for each gap-filling technique.

#### Choice of the removed months

The GRACE(-FO) solution suffers from two different types of gaps: the 11-month continuous gap and the 22 individual missing months (Fig. 1). We need to estimate the accuracy of each technique for these two specific kinds of gaps with our remove-and-restore approach. Furthermore, the noise level of the GRACE(-FO) solution varies depending on the time period. The noise level increased by a factor two after August 2016 by the end of the GRACE mission lifetime, due to the accelerometer failure of one of the two GRACE satellites (Chen et al. 2022a).

We have selected two time-slices in GRACE mission lifetime to perform the estimation of the accuracy. The first one covers the period from January 2004 to December 2011 and corresponds to GRACE mission optimal period; however, this period does not cross Swarm lifetime. The techniques based on the Swarm solution cannot be evaluated on this time-slice. The accuracy estimation for an 11-month gap during the first time-slice is referred to as ‘**Gy**’ (for GRACE-only period year-like gap), and the estimation for individual missing months during the first time-slice is referred to as ‘**Gm**’ (for GRACE-only period monthly gaps).

The second time-slice covers the period from May 2015 to August 2016. It corresponds to the period before GRACE accelerometer failure and starts one month after the beginning of the period covered by the Swarm solution. The accuracy estimation for an 11-month gap during the second time-slice is referred to as ‘**Sy**’ (for Swarm period year-like gap), and the estimation for individual missing months during the first time-slice is referred to as ‘**Sm**’ (for Swarm period monthly gaps).

Then, we randomly pick a certain number of months or one 11-month period in the whole time-slice where we can remove the GRACE products, and then apply a gap-filling technique. This testing procedure is repeated a certain number of times, and the RMS difference metrics are averaged to obtain a better estimation (keeping in mind that some techniques are computationally demanding). The dispersion is discussed with the results (section 4). For **Gm**, we randomly pick 10 months in the whole time-slice and we repeat the operation 8 times. For **Gy**, we randomly pick one 11-month gap in the whole time-slice and we repeat the operation 8 times. For **Sm**, we randomly pick 3 months in the whole time-slice and we repeat the operation 5 times. For **Sy**, we randomly pick up one 11-month gap in the whole time-slice and we repeat the operation 3 times. The number of repetitions for **Sm** and **Sy** is limited due to the reduced length of the time-segment.

## Gap-filling techniques

We consider the gap-filling techniques proposed in the literature corresponding to satellite-monitoring and interpolation methods on an Earth-wide scale. These techniques interpolate a GRACE solution with missing individual months and an 11-month gap on the whole Earth’s surface, possibly also using a Swarm solution or GLDAS model. Each technique has been implemented independently from the original article in a Python script.

### Swarm replacement

One simple technique is to replace missing GRACE(-FO) months with those from Swarm. It suffers from the noise of the Swarm data, but it can serve as a reference for more complex gap-filling techniques, which should achieve better results. In this approach, the evaluation metrics simply correspond to the RMS difference between GRACE and Swarm products.

### Constant trend annual and semiannual (CTAS) estimation

Lück et al. (2018) investigated the potential of estimating constant, trend, annual, and semiannual (CTAS) components based on a Swarm solution to bridge the gap between GRACE and GRACE-FO, technique also called “6-parameter model” or sometimes “climatology fit.” The interpolation of CTAS can also be done with the GRACE(-FO) solution. Our estimation of CTAS terms is performed by an iterative least-square fitting. The estimation of CTAS components is equivalent when done on the SH representation or on a projected grid; however, the computation is much more efficient in the SH domain as it requires fewer computations. For this reason, we estimate CTAS for Swarm and GRACE(-FO) on SH coefficients.

This technique relies on simple a priori assumptions about the solutions, suggesting that most variations in the time-series can be explained by CTAS terms. For GRACE(-FO), the evaluation metrics essentially represent the RMS variance of the signal that deviates from CTAS. Estimating CTAS with the GRACE(-FO) solution benefits from the low noise level of the products but is limited by missing data in certain months. Estimating CTAS with the Swarm solution benefits from the complete time coverage but is limited by a higher noise level.

Rateb et al. (2022) propose to use a Bayesian framework to predict missing months by estimating CTAS components as well as a long-term variability (only for individual missing months) but is not reproduced in the scope of this paper. The Bayesian estimation includes an associated uncertainty.

### Principal component analysis (PCA) with Swarm prior

Richter et al. (2021) proposed to reconstruct a GRACE-like time-variable gravity field using principal component analysis (PCA) with a Swarm solution as extra information. For this, Swarm and GRACE(-FO) solutions need to be projected onto a grid in EWH of size ($n_\lambda$, $n_\phi$, $n_t$) where $n_\lambda$ corresponds to the number of longitudes, $n_\phi$ to the number of latitudes and $n_t$ to the number of months. Based on a grid, we can create a matrix **X** with a size ($n_t$, $n_\lambda \times n_\phi$). We note this matrix **X**$_{\textrm{GRACE}}$ for the GRACE(-FO) solution and **X**$_{\textrm{Swarm}}$ for the Swarm solution. Unlike Richter et al. (2021), we normalize **X** by the cosine of the latitude associated with the row. This scales each row according to an approximation of the area of the grid point. Results are slightly better with the normalization than without, with a reduction of 0.2 cm EWH for the total RMS difference metric, according to our analysis (not shown here).

The PCA is the decomposition of **X** (equivalent to a singular value decomposition),

4 $$\begin{aligned} \textbf{X} = \textbf{USV}^T, \end{aligned}$$

where the matrix **U** with a size ($n_t$, $n_t$) contains the temporal modes (principal components), **S** is the diagonal matrix of the squared singular values, and **V** has a size ($n_\lambda \times n_\phi$, $n_t$) and contains the spatial patterns of the decomposition (eigenvectors). Each row of **U** corresponds to a temporal mode and each column of **V**$^T$ to a spatial pattern and they are ordered by the squared singular values.

Richter et al. (2021) decompose **X**$_{\textrm{GRACE}}$ using PCA to estimate **U**$_{\textrm{GRACE}}$, **S**$_{\textrm{GRACE}}$, and **V**$_{\textrm{GRACE}}$. Then, they compute a modified version of (**US**)$_{\textrm{Swarm}} = \textbf{X}_{\textrm{Swarm}}\textbf{V}^T_{\textrm{GRACE}}$ based on GRACE decomposition.

Then, $\textbf{X}_{\mathrm {Swarm~reconstruct}} = (\textbf{U}_{1:3}\textbf{S}_{1:3})_{\textrm{Swarm}}\textbf{V}^T_{1:3~\textrm{GRACE}}$ using only the first three rows of $(\textbf{US})_{\textrm{Swarm}}$ and of $\textbf{V}_{\textrm{GRACE}}$. The first three temporal modes explain $\sim 90\%$ of the signal. This cut is recommended by Richter et al. (2021) as it reduces the noise of Swarm products. Finally, we can substitute missing months with these new reconstructed ones.

Richter et al. (2021) proposed a second technique that works analogously, referred to as PCA$_{\textrm{residual}}$. The first step is to remove CTAS components fitted on the whole GRACE(-FO) period to both Swarm and GRACE(-FO) solutions. We then reconstruct the signal as described above and add back the CTAS components. We have a small divergence with Richter et al. (2021) as they remove CTAS components fitted to Swarm from the Swarm solution, while we remove CTAS components fitted on GRACE(-FO). Without this adjustment, the reconstructed products from the PCA using the Swarm prior exhibit the same bias as the Swarm solution (not shown here). Another divergence is due to the fact that we use GRACE(-FO) solution up to degree 12, while Richter et al. (2021) goes up to degree 60 and apply a Gaussian filter with a radius of 500 km. We show in Appendix C the results when we go up to degree 40.

### PCA with GLDAS prior

Gu et al. (2024) proposed an application of the PCA on SH coefficients of GRACE(-FO) products with the use of GLDAS as extra information. Hydrological loading variations of the gravity field from GLDAS are converted to SH and used along GRACE(-FO) products coefficients. Before applying the PCA, the GRACE(-FO) solution and the GLDAS model are detrended through using a least-squares fitting on the available products. Missing months in the GRACE(-FO) solution are estimated with a linear interpolation. The matrix **X** used for the PCA has a size ($2n_{l,m}$, $n_t$) where $n_{l,m}$ corresponds to the number of $C_{l,m}$ and $S_{l,m}$ coefficients ($n_{l,m}$ = 165 for a maximum degree of 12, excluding coefficients of degrees 0 and 1), and $n_t$ corresponds to the number of months. The matrix **X** is constructed by concatenating the time-series of SH coefficients from the GRACE(-FO) products and those from the GLDAS model along the rows.

**X** is decomposed with the PCA and $\textbf{X}_{\textrm{reconstruct}} = (\textbf{U}_k\textbf{S}_k)\textbf{V}^T_{k}$ using the *k* first rows of $(\textbf{US})$ and $\textbf{V}$. *k* is chosen so that the *k* first rows explain 95% of the signal variance. Reconstructed values of GRACE(-FO) SH coefficients are then replaced in the original **X** matrix for the missing months, and the reconstruction is iterated three times (Gu et al. 2024).

In Gu et al. (2024) methodology, a maximal degree of 60 is used so the matrix **X** has more rows (7434 instead of 330 for degree 12). The PCA is then applied on SH coefficients without Gaussian filtering so that north–south stripes of GRACE(-FO) solution are part of the variance. Gu et al. (2024) apply a scale factor of 3 to the hydrological model Stokes coefficients, that we do apply too, due to the noise in GRACE data and not documented (Y. Gu, personal communication, March 5, 2024). The use of the PCA with GLDAS prior technique was originally proposed to close the 11-month gap. By testing this technique also on single missing months, we go further than Gu et al. (2024)’s primary focus.

### Iterative independent component analysis (ICA)

Forootan et al. (2020) proposed an iterative reconstruction approach based on the independent component analysis (ICA). ICA is used to separate a multivariate signal into additive, independent components. This approach is known to perform better at separating signal from noise, and these signals can be more accurately related to physical phenomena (Forootan et al. 2020). Two approaches are described in their article. However, we have implemented only one (called Approach 2 in their paper), evaluated as less noisy and providing more homogenized results (Forootan et al. 2020). As for the PCA, we compute **X**$_{\textrm{GRACE}}$ and **X**$_{\textrm{Swarm}}$ matrices. Following Forootan et al. (2020), **X**$_{\textrm{ICA}} = sort([\textbf{X}^T_{\textrm{GRACE}}, \textbf{X}^T_{\textrm{Swarm}}],~\textrm{time})$ where $sort(.,~\textrm{time})$ is an operator that sorts the matrix by ascending time values. **X** rows are weighted by the inverse of the covariance matrices. **X** columns are also weighted by the cosine of the latitude (Forootan et al. (2020) mentions this possible scaling but does not mention whether it is applied).

**X**$_{\textrm{ICA}}$ is decomposed as for a PCA but using an additional matrix of rotation $\textbf{R}$, so that $\textbf{V}\textbf{R}$ columns are as independent as possible,

5 $$\begin{aligned} \textbf{X}_{\textrm{ICA}} = \textbf{URR}^T\textbf{SV}^T \end{aligned}$$

where $\textbf{R}$ is computed using the JADE ICA algorithm (Cardoso 1999).

As for the PCA, we compute $\hat{{\textbf {X}}}= \textbf{U}_{k}\textbf{R}_{k}\textbf{R}_{k}^T\textbf{S}_{k}\textbf{V}_{k}^T$ using only the first *k* rows. *k* is chosen so that it represents a part of the variance of **X**$_{\textrm{ICA}}$. The values of $\hat{{\textbf {X}}}$ that correspond to the Swarm rows replace the initial values of **X**, and then we loop on the ICA decomposition. This iteration stops when the total RMS difference between modes is less than 10$^{-6}$.

It is worth mentioning that Forootan et al. (2020) does not exactly use the same GRACE(-FO) and Swarm solutions. They use SH products up to degree 96 for GRACE(-FO) and up to degree 40 for Swarm. They apply an isotropic Gaussian filter with the radius of 500 km for GRACE(-FO) and a Gaussian smoothing filter with 1000 km half-wavelength radius for Swarm (Jekeli 1981). To deal with this difference, we reduce *k*, the used rows for the reconstruction, so that the part of the variance explained with the PCA is 90% (instead of 95% in Forootan et al. (2020)). By applying this change to the explained variance, the total RMS difference metric is reduced by 0.5 cm EWH, corresponding to a significant improvement (not shown here). By using more variance, the ICA prediction converges to the Swarm solution.

### Iterative singular spectrum analysis (SSA)

Yi and Sneeuw (2021) proposed a gap-filling technique using singular spectrum analysis (SSA) on SH coefficients of a GRACE(-FO) solution. In the following, our notations differ from that of Yi and Sneeuw (2021). Each SH coefficient is transformed into a uniformly sampled time-series, $[z_1,~z_2,~\ldots ,~z_{n_t}]$, with $n_t$ the total number of months (= 249) and where months with no data are set to 0. In the SSA decomposition, this time-series can be accounted for to build the trajectory matrix **Y**,6where $L=n_t+1-M$ is the number of columns, arbitrarily chosen. It corresponds to the number of months used in the SSA.

The SSA consists of decomposing **Y** with a PCA in order to obtain *L* eigenvectors **V**$^Y$. Then, the spatio-temporal principal components are

7 $$\begin{aligned} PC^k(t) = \sum \limits _{i=1}^{L}z_{t + i -1}.\textbf{V}^Y_{i,k}~, \end{aligned}$$

with $t \in [1, M]$, $k \in [1, L]$ and $\textbf{V}^Y_{i,k}$ corresponds to the *i*-th row and *l*-th column of **V**.

The time-series $\textbf{V}^Y$ and *PC* allow to create the reconstructed components, ordered on *k* in descending order of their singular value,

8 $$\begin{aligned} RC^k(t) = {\left\{ \begin{array}{ll} \frac{1}{t} \sum \limits _{i=1}^{t} PC^k(t - i + 1).\textbf{V}^Y_{i,k}& \text {if } 1 \le t \le L-1\\ \frac{1}{L} \sum \limits _{i=1}^{L} PC^k(t - i + 1).\textbf{V}^Y_{i,k}& \text {if } L \le t \le n_t - L+1\\ \frac{1}{n_t - t + 1} \sum \limits _{i=L-n_t+t}^{L} PC^k(t - i + 1).\textbf{V}^Y_{i,k}& \text {if } N-L+2 \le t \le n_t \end{array}\right. } \nonumber \\ \end{aligned}$$

The original time-series is then reconstructed as $z_t = \sum \nolimits _{k=1}^L RC^k(t)$, with $t \in [1, n_t]$. By choosing an arbitrary *K*, one can compute the time-series of the original SH coefficient on a limited number of reconstructed components to remove the noisier parts.

In Yi and Sneeuw (2021), an iterative approach is applied where the SSA estimates months without data. It starts with $K=1$ and when the estimation of the month is stable enough, a new iteration starts with $K=2$. *K* increases in a second iterative loop up to $K_\textrm{max}$ chosen arbitrarily for the SH coefficient. Yi and Sneeuw (2021) apply this technique twice with different *M* and $K_\textrm{max}$ values, first, to estimate individual missing months and second, to fill the 11-month gap. For the first round, the parameters are set to $M=24$, corresponding to a context of 2 years around the missing month, and $K_\textrm{max}=12$. For the second round, the optimal parameters are $M=48$ and $K_\textrm{max}=7$. The authors also expose a cross-validation algorithm to obtain the best *M* and $K_\textrm{max}$ for each coefficient (not considered in our study, as computationally demanding).

Our implementation of the SSA algorithm is based on the MATLAB code of Yi and Sneeuw (2021) translated into a Python code and computationally optimized for Python.

### Improved multichannel SSA (MSSA)

Wang et al. (2021) proposed to fill the gap by applying the improved multichannel SSA (MSSA). This technique is close to SSA but uses the information of all SH coefficients during the reconstruction. As with SSA, each coefficient is transformed into a uniformly sampled time-series. However, once the trajectory matrix **Y**$_{l,m}$ is constructed for each SH coefficient of degree *l* and order *m*, **Y**$_{\text {MSSA}}$ is the concatenation of all the **Y**$_{l,m}$ along the columns with a size of (*M*, $n_{l,m}$). Where *M* is the arbitrarily chosen number of rows and $n_{l,m}$ is the number of SH coefficients used ($n_{l,m}$=165).

Then, **Y**$_{\text {MSSA}}$ is decomposed using a singular value decomposition on the covariance matrix detailed in Wang et al. (2021), considering the missing data. The coefficients are recomposed similarly to the SSA, following Wang et al. (2021). This non-iterative reconstruction predicts each SH coefficient time-series. The two parameters of the improved MSSA are defined with $M=60$ and $K_\textrm{max}=12$. The time-window of the method corresponds to 60 months and the reconstruct order of 12 has been validated by an analysis of the w-correlations of the first modes (see Fig. 1 from Wang et al. (2021)). As for the PCA with hydrological prior, imposing a maximal degree of 12 instead of 60 for Wang et al. (2021) reduces the ability of MSSA to extract common variance between SH coefficients.

Gauer et al. (2023) proposes to use MSSA to predict missing months and to filter GRACE(-FO) solutions but is not reproduced in the scope of this paper. They used an iterative MSSA to replace missing data combined with spatial filtering. However, the channel components of the MSSA from Gauer et al. (2023) correspond to different SH products projected onto a grid, and the technique is applied to the grid. Given our decision to use only products from COST-G solution, adapting Gauer et al. (2023) to our assessment would make it correspond to the SSA technique from Yi and Sneeuw (2021) applied to a grid.

### Auto-regressive (AR)

Lenczuk et al. (2022) proposed a remove-and-restore technique combined with an auto-regressive (AR) prediction. For this, GRACE(-FO) solution needs to be projected on a grid in EWH with a size ($n_\lambda$, $n_\phi$, $n_t$). The GLDAS hydrological model is also used as projected on a grid. The first step is to subtract from GRACE(-FO) product the hydrological signal with GLDAS and then to remove the remaining CTAS components.

The residuals time-series of each point of the grid, $[r_1,~r_2,~\ldots ,~r_{n_t}]$, are needed to estimate an AR model where $r_i$ is expressed as

9 $$\begin{aligned} r_i = a_1r_{i-1} + a_2r_{i-2} + \cdots + a_Mr_{i-M} + \varepsilon ~, \end{aligned}$$

where $a_1,~a_2, \ldots ,~a_M$ are the AR coefficients, *M* is the AR order that is chosen arbitrarily, and $\varepsilon$ is the residual that statistically corresponds to a white noise.

AR coefficients are estimated using the following equation:10where $c_k = \frac{1}{n_t}\sum _{i=1}^{n_t-k}r_i r_{i+k}$ is the biased auto-covariance estimate (Lenczuk et al. 2022).

**Table 1:** Summary of the different gap-filling techniques applied in this paper

Technique	Apply to	Residual	Extra data	References
Swarm replacement	SH	No	Swarm	
CTAS GRACE	SH	No		
CTAS Swarm	SH	No	Swarm	Lück et al. (2018)
PCA Swarm	Grid	No	Swarm	Richter et al. (2021)
PCA Swarm residual	Grid	Yes	Swarm	Richter et al. (2021)
PCA GLDAS	SH	No	GLDAS	Gu et al. (2024)
PCA GLDAS residual	SH	Yes	GLDAS	
ICA	Grid	No	Swarm	Forootan et al. (2020)
ICA residual	Grid	Yes	Swarm	
SSA	SH	No		Yi and Sneeuw (2021)
SSA residual	SH	Yes		
MSSA	SH	No		Wang et al. (2021)
MSSA residual	SH	Yes		
AR	Grid	Yes	GLDAS	Lenczuk et al. (2022)

From the AR model, Lenczuk et al. (2022) predict recursively the missing months in the 11-month gap using an AR order of $M=24$. The AR model is used in a classic forward approach and a backward approach where the *i* index from equation (9) is incremented. The forward and backward predicted time-series are artificially modified so that they join each other in the middle of the gap. Before the middle, the forward time-series is used and after the middle, the backward one (Lenczuk et al. 2022).

A few things need to be mentioned here. The technique is proposed to interpolate the 11-month gap on continental regions only and is not designed to fill the individual gaps. It has been modified to fill individual gaps by training forward and backward predictions on the whole time-series. Missing months are considered as zero for the estimation of the auto-covariance and when they are needed in the interpolation. We also employ the AR technique to predict the time-series of grid points over the oceans. It was originally meant to be handled on the GRACE(-FO) with a spatial resolution of the order of a few hundred kilometers. Its usage for a product projected on a grid from a SH product truncated at degree 12 is at the border of the technique’s original purpose. The GLDAS hydrological model used by Lenczuk et al. (2022) is GLDAS Noah 2.1; however, we use GLDAS Catchment Land Surface Model 2.1. This modification does not significantly change the reconstruction (not shown here).

### Summary of the techniques

It is important to emphasize that all the tested techniques were slightly modified and taken out of their initial design framework in order to be comparable in our benchmark.

Based on the residual approach from Richter et al. (2021), we propose to evaluate PCA, ICA, SSA, and MSSA techniques with an analog residual approach. It consists of removing CTAS components fitted on GRACE(-FO) products for the whole time period (2002–2022) to both GRACE(-FO) and Swarm or GLDAS (if used) products. Then, we reconstruct the signal via the technique and we add back the CTAS components. With this twist, the technique is just asked to predict changes that are not trend, annual, and semi-annual signals and less information is needed to predict the expected time-series.

All the gap-filling techniques are summarized in the Table 1.

Variance decomposition techniques (PCA, ICA, SSA, and MSSA) involve defining a parameter (*k* or $K_\textrm{max}$, the number of rows used for the reconstruction of the signal) linked with the part of the variance explained in the decomposition. Choosing this parameter requires an understanding of the signal being decomposed. The portion of the variance used depends on the noise level of the time-series. Using too much variance implies that some noise is used for the reconstruction, while using not enough variance implies missing some true variation in the signal. It is even more complex for decomposition with additional data (GLDAS model or Swarm products) where using a too large portion of the variance will make the techniques reconstruct with additional noise coming from the extra data. However, GRACE(-FO) products are provided only with uncertainties corresponding to the formal errors of the inversion from orbit arcs to SH coefficients. The formal errors are not related to the uncertainty of the various measurement components of GRACE(-FO) missions (K-band Ranging System, accelerometers, global navigation satellite system positioning). As a user, the choice of the portion of the variance to use in the variance decomposition is already a deliberate commitment.

## Results

### Assessment workflow

All the presented techniques have been evaluated via our benchmark methodology to estimate the total RMS difference with the GRACE months (truncated at degree 12), removed-and-restored projected to a grid in cm EWH (Table 2). Techniques partially based on the Swarm solution are not evaluated with **Gm** and **Gy** procedures as the corresponding time period contains no Swarm products. As the metric estimation is repeated and averaged, it includes the standard deviation of the total RMS differences. This standard deviation can reach up to 0.2 cm EWH. In the following, we assume that 0.2 cm EWH is the threshold for statistically significant differences. As a validation of our evaluation, we estimated the total RMS difference with the GRACE months removed-and-restored on continent and ocean where no anomalies are found (Appendix B, Table 3).

**Table 2:**
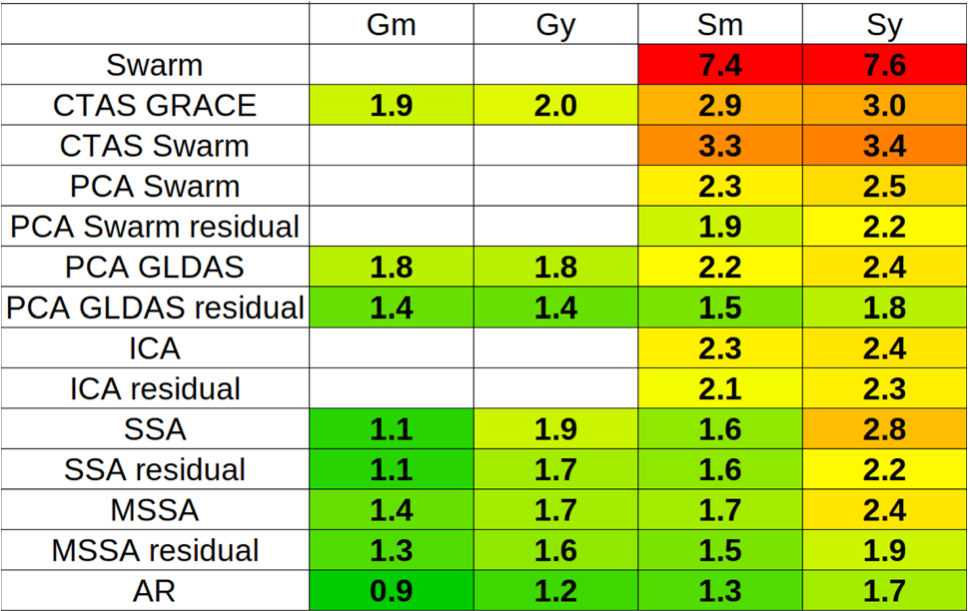
Evaluation of the accuracy of the gap-filling techniques measured as the total RMS difference with the GRACE months removed-and-restored projected to a grid in cm EWH. GRACE and Swarm products are truncated at degree 12

The gap-fill techniques employing Swarm products to substitute for GRACE(-FO) products have the higher total RMS differences with values of 7.4 cm EWH for **Sm** and 7.6 cm EWH for **Sy**. This is due to the higher noise level of the Swarm products compared to GRACE(-FO). Total RMS differences are higher for all the techniques after 2015 for **Sm** and **Sy**. Despite that the techniques demonstrate a certain performance to predict GRACE(-FO)-like time-series, they are not able to predict the noisy behavior at the end of GRACE mission, resulting in an increase of the total RMS differences. The CTAS estimation performs better with fit on GRACE(-FO) time-series than with fit on Swarm time-series (the missing months in GRACE(-FO) time-series perturb less the estimation than the noise of Swarm products). It is worth mentioning that the total RMS difference with CTAS using GRACE(-FO) solution, of 2.0 cm EWH on the 2004–2011 period, is less than twice larger than the total RMS difference with other techniques.

Residual approaches exhibit better performance compared to their non-residual counterparts. However, the improvement attributed to the residual approach is not statistically significant ($\le$ 0.2 cm EWH) for ICA, SSA and MSSA. In the residual approach, the technique is asked to predict changes that exclude trend, annual and semi-annual signals. Because of this, less information is needed to predict the expected time-series. However, the part of the variance corresponding to noise is also increased as we reduce the coherent signal by removing CTAS. The parameters of ICA, SSA and MSSA (the number of iterations, the number *k*/$K_\textrm{max}$ of principal components that explain a part of the variance and the size *M* of the context window) have not been fine-tuned specifically for the residual approach, and it is plausible that such fine-tuning could improve their score. Using the variance decomposition techniques (PCA, ICA, SSA and MSSA) requires an understanding of the signal to find optimal parameters. For instance the choice of the parameters *k*/$K_\textrm{max}$ is contingent upon the noise level of the data. Moreover, as the quality of GRACE(-FO) changes through time, the optimal values of the parameters are also evolving. This explains, in addition to the noise, the degraded score of the SSA technique on the **Sy** procedure.

According to our assessment criteria, the AR technique is the one with the best score in terms of total RMS difference between the predicted monthly products and the removed ones. AR scores are between 0.9 and 1.7 cm EWH with the four procedures. The difference is not statistically significant with SSA on individual months (**Gm** and **Sm**) but is statistically significant for 11-month gap (**Gy** and **Sy**). The AR process requires one parameter, the auto-regressive order, which defines the spectral content of the predicted signal. Its optimal value will hence be influenced by the filtering of data and their noise. The AR technique is also using a double residual approach by first removing hydrological variations from GLDAS model and then removing CTAS. The AR score is two to three times larger than the GRACE(-FO) solution uncertainty. The score of a gap-filling technique cannot be interpreted if its value is below the solution uncertainty. The lower bound of the GRACE(-FO) solutions uncertainty at degree 12 has been estimated at 0.4 cm EWH under similar conditions, using Gaussian spatial filtering with a radius of 1200 km, which cancels the SH amplitude beyond degree 12 (Lecomte et al. 2023).

The total RMS differences are higher for the techniques using Swarm solution than for the others. They are the techniques (CTAS Swarm, PCA Swarm and ICA) with the highest RMS differences on ocean (Appendix B, Table 3). While they benefit from additional information from the complementary dataset, these techniques also suffer from the noise of Swarm products.

**Fig. 3:**
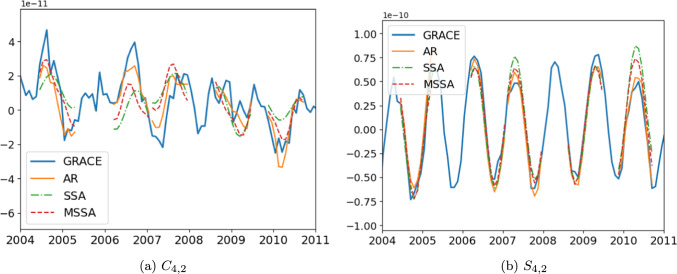
SH coefficient time-series from GRACE between 2004 and 2011 and 11-month prediction from AR, SSA and MSSA techniques predicted with the remove-and-restore workflow

The understanding of the RMS differences of the techniques can be strengthened by examining their behavior over specific SH coefficients. The coefficient $S_{4,2}$ does have a strong annual cycle with amplitude variation (Fig. 3). The coefficient $C_{4,2}$ lacks an annual cycle but displays inter-annual behavior that is irregular and not dominated by any particular sinusoidal cycle (Fig. 3). Examples of five 11-month predictions from AR, SSA and MSSA techniques on $C_{4,2}$ and $S_{4,2}$ explain the score differences between the techniques. AR demonstrates adaptability to amplitude variation in the $S_{4,2}$ annual cycle in 2006, 2007 or 2010, while SSA and MSSA are less successful. Predictions for $C_{4,2}$ further highlight why AR total RMS differences are smaller than those of MSSA, which are also smaller than those of SSA in the **Gy** procedure. SSA predictions are smoother over the 11-month gap, while AR and MSSA are able to be closer to the behavior of $C_{4,2}$. As the SSA technique is run twice, once on individual months with specific parameters and another one on the 11-month segment with other parameters, it may explain the difference of scores between monthly (**Gm**, **Sm**) and 11-month (**Gy**, **Sy**) procedures.

We have also conducted our assessment methodology on GRACE products truncated at degree 40 and Gaussian filtered with a radius of 400 km (Appendix C). For the techniques using Swarm solution, they were used with a truncation at degree 12. Total RMS differences scores given in Appendix C, Table 4, are statistically close to the ones given in previous studies when available. The **Gy** procedure gives a score of 1.9 cm EWH for MSSA that is slightly smaller than the score of 2.1 cm EWH in Wang et al. (2021). The **Gy** procedure gives a score of 2.1 cm EWH for PCA using GLDAS prior that is slightly larger than the score of 2.05 cm EWH in Gu et al. (2024).

### Spatial RMS differences of the gap-filling techniques

**Fig. 4:**
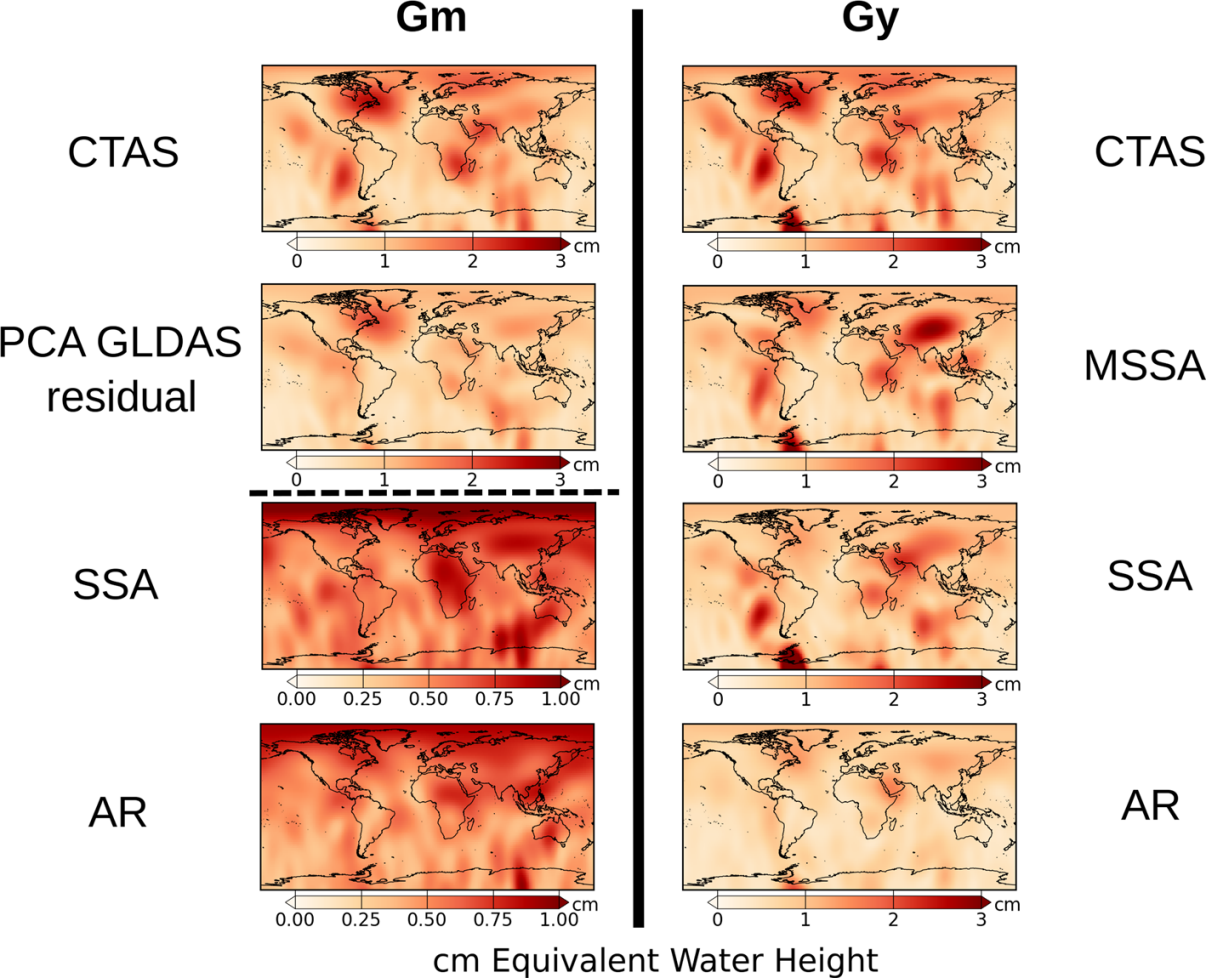
Spatial RMS differences in cm EWH on removed-and-restored months for CTAS based on GRACE(-FO), PCA with GLDAS prior residual, MSSA, SSA and AR techniques. The left column corresponds to remove-and-restore approach with **Gm** procedure and the right column with **Gy** procedure. The scale of the two maps on the bottom-left is modified for readability

We show here that examining the spatial distribution of RMS differences for each assessment procedure is preferable (Fig. 4). When considering CTAS fitted on GRACE(-FO) solution, spatial errors are similar between **Gm** and **Gy**. This is expected as the prediction of CTAS remains consistent between the two procedures. Consequently, the spatial RMS differences maps for CTAS depict the spatial amplitude of the GRACE(-FO) products for variations excluding trends and annual or semi-annual cycles. These non-CTAS time variations are noticeable on the East coast of North America, the West coast of South America, around the Filchner-Ronne Ice Shelf in Antarctica, Indian Ocean, South of Africa, Middle of Asia, and Northern Europe and Russia. These locations with large differences also coincide with areas exhibiting larger RMS differences for other techniques. This is illustrated in Fig. 4 with the PCA using GLDAS prior and residual approach for the **Gm** procedure and with the MSSA or SSA for the **Gy** procedure. These two sub-figures show similar spatial patterns to the CTAS ones but with lower amplitudes, indicating that non-CTAS time variations are hard to predict using these techniques. The AR spatial distribution of RMS differences for the **Gy** procedure exhibits a pattern consistent with non-CTAS time variations but with a lower amplitude than other techniques.

The amplitude of the RMS differences is lower with SSA and AR for the **Gm** procedure (please note that the color scale stops at 1 cm, while for other subplots it stops at 3 cm in Fig. 4). While these two techniques outperform the others for individual missing months, we aim to facilitate the discussion of the spatial distribution of RMS differences. Some areas with non-CTAS time variations, such as the Indian Ocean, show larger RMS differences. The geographic North Pole region appears problematic for SSA and AR, potentially due to specific SH coefficients, although the degree 1 coefficients are set to zero. This pattern might also be present in other techniques but hidden by the larger color-map scale. Despite visible spatial patterns, RMS differences are more spatially homogeneous than those of the other techniques.

Spatial RMS differences for the **Sm** and **Sy** procedures are predominantly influenced by sectorial SH patterns at low degrees, resulting in significant north–south errors. This effect is attributed to the higher noise levels observed between May 2015 and August 2016. Not much information can be extracted for these spatial differences on the period, leading to their omission from Fig. 4 for **Sm** and **Sy**. Each technique struggles in predicting the noisy behavior of GRACE products during this period, reflected in total RMS difference scores ranging between $\sim 1.5$ and 2.5 cm EWH.

### Spectral analysis of the techniques

**Fig. 5:**
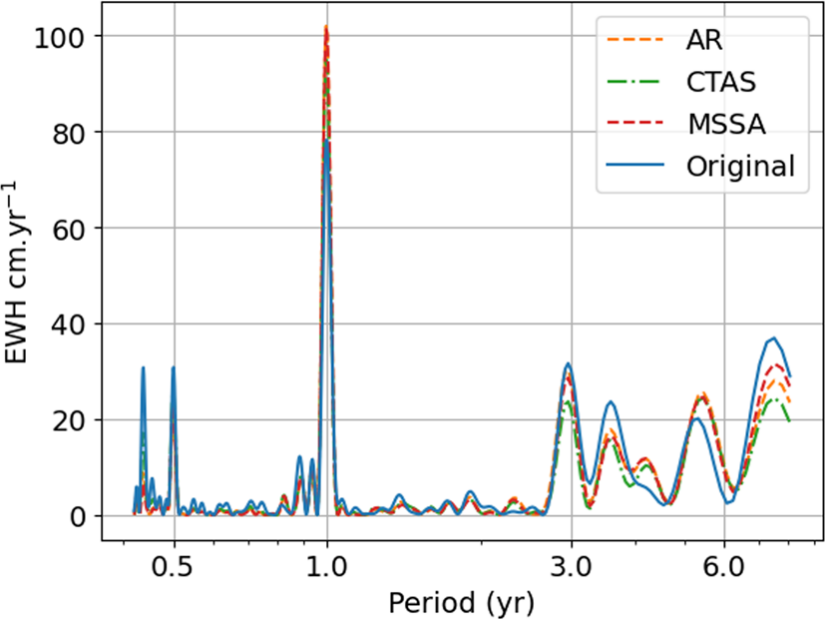
Lomb-Scargle periodogram of a GRACE(-FO) time-series with gaps (blue solid line) and the same GRACE(-FO) solution gap-filled with CTAS, AR and MSSA techniques (respectively, in green dash-dotted, orange dashed and red dashed lines). The time-series correspond to the gravity field variations at a point located at 45$^{\circ }$N, 19$^{\circ }$W in the Atlantic ocean

Gap filling a time-series alters its spectral content in most cases. The uneven sampling of the gravity-field time-series introduces biases in trend and inter-annual cycle estimations (Santamaría-Gómez and Ray 2021), which can be demonstrated by a periodogram analysis (Fig. 5). The Lomb-Scargle periodogram of gravity field variations at a point located at 45$^{\circ }$N, 19$^{\circ }$W in the Atlantic Ocean reveals both annual oscillations and inter-annual power (location selected for its rich spectral content). We show here the periodogram of the time-series gap-filled with CTAS (based on GRACE(-FO)), AR and MSSA techniques. CTAS being the simplest and AR and MSSA achieving the best scores for 11-month gap. The periodograms of the other techniques are similar to the showcased ones, and our selection does not alter the presentation of the results and the discussions. A comparison between the periodogram of the GRACE(-FO) solution with gaps and the periodograms of the gap-filled time-series indicates an underestimation of power at the annual period by 20%. The periodogram power is misestimated at inter-annual scales. The periodograms derived from the gap-fill using CTAS, AR and MSSA techniques show overall agreement, with exceptions noted for the 3-yr period where the MSSA spike is closest to the original time-series spike and at the 7.5-yr period.

### Comparison of predictions with low Earth’s orbit satellites products

**Fig. 6:**
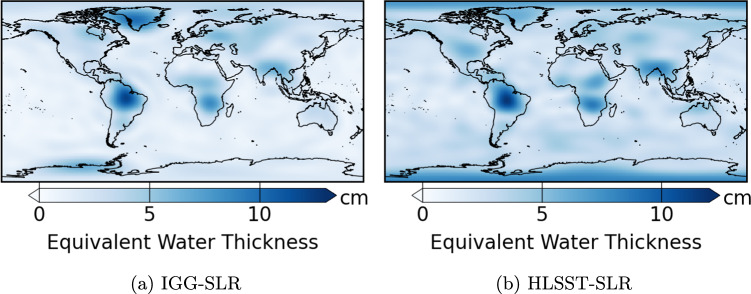
Spatial RMS differences on GRACE(-FO) missing months between predictions from the AR technique and two low Earth’s orbit satellites, IGG-SLR in (a) and HLSST-SLR in (b)

To enhance the assessment of the gap-filling techniques, we compare their predictions with two existing monthly time-variable gravity field solutions based on low Earth’s orbit satellites orbits and presented in the introduction. The solution from Löcher and Kusche (2020), corresponding to the Ensemble Mean solution, is denoted as IGG-SLR. The solution from Weigelt (2019), corresponding to the 2023 solution with an applied Kalman filter, is denoted as HLSST-SLR. IGG-SLR and HLSST-SLR with a maximum SH degree 12 have been compared with the predictions of the different gap-filling techniques on GRACE(-FO) missing months. The used metric is the total RMS difference weighted by latitude, and spatial maps of these RMS differences are produced (Fig. 6).

The total RMS difference between IGG-SLR and the gap-filling techniques is stable, ranging from 2.6 to 3.0 cm EWH. Over the continents, the total RMS difference ranges from 4.2 to 4.4 cm EWH, while over oceans, it varies between 1.6 and 1.7 cm EWH. This stability in total RMS difference with the various techniques suggests that the noise level of IGG-SLR is larger than the estimated accuracy of the gap-filling techniques. The variations in total RMS difference between continents and oceans may be due to the dependency of IGG-SLR error on signal amplitude because of its construction based on empirical orthogonal functions. A similar pattern is observed for HLSST-SLR with total RMS difference ranging between 3.3 and 3.6 cm EWH. Over continents, difference between HLSST-SLR and the gap-filling techniques ranges from 4.6 to 4.9 cm EWH and between 2.6 and 2.8 cm EWH over oceans. The stability in total RMS difference with the panel of gap-filling techniques indicates that the noise level in HLSST-SLR is larger than the estimated accuracy of the gap-filling techniques. The total RMS difference can be reduced to 3.1-$-$3.4 cm EWH by using a Gaussian filter with a radius of 700 km, similarly to Weigelt (2019).

The techniques with the closest scores to the low Earth’s orbit solutions are AR and PCA with GLDAS prior and residual approach. Their total RMS differences correspond to the lowest values between all the techniques. Spatial maps of RMS differences are quite similar for each technique. As an example, spatial differences are presented for the AR technique (Fig. 6). HLSST-SLR appears slightly noisier than IGG-SLR over oceans. Areas with the largest RMS differences correspond to regions with large gravity field variations. Additionally, IGG-SLR shows more differences on Greenland compared to HLSST-SLR, while HLSST-SLR exhibits more differences at the poles.

## Discussions and conclusions

Different gap-filling techniques have been compared in a benchmark using a remove-and-restore methodology. We have considered gap-filling techniques corresponding to satellite-monitoring and interpolation methods on a global scale. We have applied these techniques to the COST-G SH solution up to degree 12 and to degree 40 with a Gaussian filter of radius 400 km. The evaluation metrics used to estimate the errors associated with each technique are the RMS differences between the removed-and-restored months. According to such metrics, AR is the most accurate to estimate both the individual missing months and the 11-month gap. SSA is the second most accurate technique to estimate individual months, while MSSA is the second most accurate to estimate the 11-month gap. Additionally, the most accurate technique to estimate individual months and the 11-month gap using the Swarm solution is PCA with a residual approach.

Based on the metrics, we have obtained a ranking of the presented methods. Nevertheless, other criteria might be taken into account when choosing the gap-filling technique to apply to a GRACE(-FO) solution. For example, one might consider the criteria of simplicity and computational speed. Depending on the weights assigned to the decision-making criteria, the CTAS technique might be chosen for its easy implementation and fast computational time. However, CTAS estimations are less accurate than the AR technique, with a total RMS difference less than twice as large. One might also consider a criteria based on the ability to monitor sub-monthly “non-predictable” events, such as earthquakes. In this case, the inclusion of some additional data is needed, with a data-driven method (not studied in this paper) or with a satellite-monitoring method.

We note that the noise levels observed in the IGG-SLR and HLSST-SLR products are higher than the assessed accuracy of the gap-filling techniques. They do not rely on direct observations from GRACE(-FO) during missing-months but instead use observations from other low Earth’s satellites orbits. In contrast, the gap-filling techniques deduce missing months from GRACE(-FO) observations, and even with some uncertainty, they align more closely to the original data. We point out that the IGG-SLR and HLSST-SLR solutions have other advantages, as they are based on real data, allowing them to account for hardly predictable events (e.g., earthquakes). IGG-SLR also has the advantage of providing a time-series that goes back to 1993.

In the future, global interpolation methods for a GRACE(-FO) solution should be compared to existing methods to assess their reliability and their relevance. Variance decomposition techniques achieving more adaptability to the data noise level changing over time might offer a pathway to achieve better scores. The extensive family of auto-regressive models suggests that alternatives can be explored, such as auto-regressive-moving-average or auto-regressive integrated moving-average models. Additionally, alongside interpolation techniques, new gravity-field solution made by combining tracking of satellite orbits with better accuracy may emerge as alternatives for accessing low-degree variations in the gravity field. Nonetheless, our capacity to interpolate missing months in GRACE(-FO) solution is also limited by the product-accuracy itself. The accuracy of our predictive techniques cannot achieve a lower value than the uncertainty of GRACE(-FO) products. Lecomte et al. (2023) estimated the lower bound of this uncertainty at 0.4 cm EWH in a similar condition with a Gaussian spatial filtering of radius 1200 km that cancels the SH higher than degree 12.

To go further in the evaluation of the different gap-filling techniques, other approaches might be considered too. We can use a synthetic solution of the time-variable gravity field to better separate the errors introduced by the interpolation techniques from those inherent in the GRACE(-FO) data. We can take advantage of known properties of the products such as RMS values over the oceans to estimate the resulting artificial noise created in the prediction of the missing months. Investigation of cross-validation using external datasets is also a path to explore. We can compare the estimates from the techniques based on their capability to predict the mean sea level over several months or on specific large hydrological basins. The taken-side of this study is a clear choice to evaluate the gap-filling techniques ability to predict GRACE-like products (without considering their noise level variability through time). For hydrological basins, the assessment can be made with, for example, water-balance variables available from climate models or field measurements. Such alternative assessment method would verify the performance of gap-filling techniques in predicting mass changes derived from climate models or field measurements.

The obtained results highlight the challenges and difficulties created by temporal gaps in a gravity-field solution. For example, spectral analysis methods like periodograms are sensitive to data gaps. Ensuring continuity in the time-variable Earth’s gravity field measurements is crucial. While no discontinuities have been detected between GRACE and GRACE-FO products so far (e.g., (Landerer et al. 2020; Velicogna et al. 2020)), this might partly explain the non-closure of the global mean sea-level budget after 2016 (Barnoud et al. 2021). To ensure observational continuity between satellite gravimetry missions, inter-comparison periods are preferable. The end of the GRACE-FO mission is scheduled for 2028 (Landerer 2023) and, hopefully, the following GRACE-Continuity mission will be launched before then. The Mass Change and Geoscience International Constellation (MAGIC) (Heller-Kaikov et al. 2023), by its construction, is the swagger of inter-comparison between satellite gravimetry missions. However, the future beyond MAGIC remains uncertain but might be illuminated by the development of an operational mission for the time-variable Earth’s gravity field measurements.

## Figures and Tables

**Table 3:**
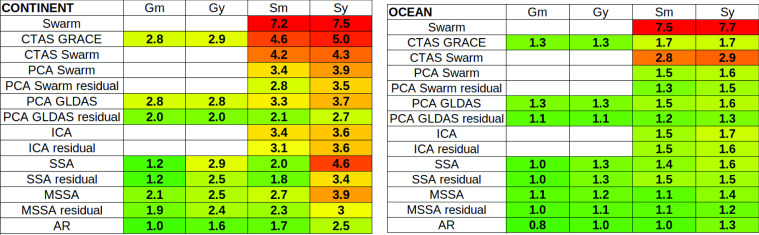
Evaluation of the accuracy of the gap-filling techniques measured as the total RMS difference with the GRACE months removed-and-restored projected to a grid in cm EWH over continents (left table) and oceans (right table)

**Table 4:**
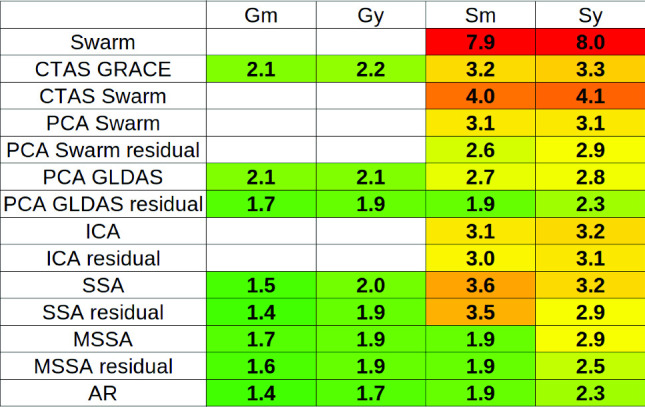
Evaluation of the accuracy of the gap-filling techniques measured as the total RMS difference with the GRACE months removed-and-restored projected to a grid in cm EWH. GRACE products are truncated after degree 40 and smoothed with a Gaussian filter of radius 400 km. Swarm products are truncated after degree 12

## Data Availability

GRACE and GRACE-FO missions are sponsored by the National Aeronautics and Space Administration and the Deutsches Zentrum für Luft-und Raumfahrt. GRACE and GRACE-FO Level-2 temporal solutions were obtained from http://icgem.gfz-potsdam.de for COST-G products. Release 01 has been used for the GRACE period and Release 02 for the GRACE-FO period. GLDAS Catchment Land Surface Model 2.1 can be downloaded from https://daac.gsfc.nasa.gov/datasets.

## Appendix A Acronyms

AIUB: Astronomical Institute of the University of Bern
AOD1B: atmosphere and ocean de-aliasing Level 1B
ASU: Astronomical Institute Ondrejov
AR: auto-regressive
COST-G: International Combination Service for Time-variable Gravity Fields
CSR: Center for Space Research
CTAS: constant, trend, annual, and semiannual
ESA: European Space Agency
EWH: Equivalent water height
GFZ: German Research Centre for Geosciences
GLDAS: Global Land Data Assimilation System
GPS: Global Positioning System
GRACE: Gravity Recovery and Climate Experiment
GRACE-FO: GRACE-Follow On
GRGS: Groupe de Recherche de Géodésie Spatiale
ICA: Independent component analysis
IfG: Institute of Geodesy Graz
JPL: Jet Propulsion Laboratory
LUH: Leibniz Universität Hannover
MAGIC: Mass Change and Geoscience International Constellation
MSSA: Multichannel SSA
OSU: Ohio State University
PCA: Principal component analysis
RMS: Root-mean-square
SH: Spherical harmonics
SLR: Satellite laser ranging
SSA: Singular spectrum analysis
TWS: Terrestrial water storage
VCE: Variance component estimation

## Appendix B RMS differences over continents and oceans

All the techniques have been evaluated with our methodology to estimate the total RMS difference with the GRACE months (truncated at degree 12) removed-and-restored, projected to a grid in cm EWH for continental and oceanic areas.

The hierarchy of the technique scores remains unchanged for continental and oceanic areas. The AR technique has the best score in terms of total RMS difference for both. For continents, the scores have increased because the amplitude of the signal is larger on average. For oceans, the scores have increased because the amplitude of the signal is smaller on average.

Swarm replacement technique shows similar total RMS difference on continents and oceans, indicating poor results due to the noise of the Swarm products, as expected. The other techniques show a higher total RMS difference over continents than over oceans within a scale factor of difference. This can be interpreted as the techniques struggling more on areas with higher-amplitude signals, which is an expected behavior.

## Appendix C Assessment of gap-filling techniques for degrees up to 40

To extend our assessment of the techniques, we have estimated the total RMS difference with the GRACE months removed-and-restored methodology, truncated at degree 40 (instead of 12) and Gaussian filtered with a radius of 400 km. The total RMS difference over oceans and continents has also been tested and agreed with the results obtained in Appendix B (not shown here).

The hierarchy of the technique scores remains unchanged, with AR having the best score in terms of total RMS difference between predicted monthly products and the removed ones. Each score is however increased compared to the assessment of the techniques with truncation at degree 12. This increase is expected, as the products contain signals between degrees 12 and 40 that are added, even though a part of this signal is filtered. Nevertheless, the increase in score is not uniform across the techniques. The largest increases correspond to the scores of the techniques that use Swarm solution. This is anticipated since they do not benefit from additional information from the Swarm products beyond degree 12 Teixeira da Encarnação et al. (2020). The score of SSA for the **Sm** procedure is relatively high, possibly indicating a non-optimal choice of the *M* and $K_\textrm{max}$ parameters, which can be further optimized through cross-validation following the approach proposed by Yi and Sneeuw (2021).

## Code Availability

The Python 3.8 code used for this publication is based on a Github project by Tyler Tsutterley (https://github.com/tsutterley/read-GRACE-harmonics, https://doi.org/10.5281/zenodo.8075728). The adapted version can be found on https://github.com/hulecom/read-GRACE-harmonics repository. JADE algorithm from Gabriel Beckers https://github.com/gbeckers/jadeR. SSA original code https://doi.org/10.18419/darus-807.
